# Clinical characteristics and outcomes of newly diagnosed patients with human immunodeficiency virus-associated Burkitt lymphoma: the Central and Western China AIDS lymphoma league 002 study (CALL-002 study)

**DOI:** 10.1186/s13027-023-00559-y

**Published:** 2023-12-05

**Authors:** Jinrong Zhao, Haiyan Min, Yunhong Huang, Yaokai Chen, Min Wang, Lirong Xiao, Guo Wei, Yan Wu, Yao Liu, Wei Zhang

**Affiliations:** 1 Department of Hematology, Peking Union Medical College Hospital, Peking Union Medical College, Chinese Academy of Medical Sciences, 1 Shuaifuyuan Wangfujing Street, Beijing, 100730 China; 2 Yunnan Provincial Hospital of Infectious Diseases, Kunming, 650301 China; 3 Affiliated hospital of guizhou medical university, Guiyang, 550004 China; 4 Chonqging Public Health Medical Center, Chongqing, 400036 China; 5 The First Hospital of Changsha, Changsha, 410011 China; 6 Henan Infectious Disease Hospital, The Sixth People’s Hospital of Zhengzhou, Zhengzhou, 450015 China; 7 Public health clinical center of Chengdu, Chengdu, 610066 China; 8 Department of Hematology Oncology, Chongqing University Cancer Hospital, Chongqing, 400030 China

**Keywords:** AIDS, Lymphoma, Burkitt, Outcome, China, Human immunodeficiency virus

## Abstract

**Background:**

Despite the introduction of combined antiretroviral therapy, the clinical outcomes of HIV-associated Burkitt lymphoma (BL) remain poor.

**Methods:**

To evaluate the clinical characteristics, prognostic factors, and outcomes of HIV-associated BL, we conducted a retrospective analysis of patients from multiple centers in China.

**Results:**

The study included 41 patients from 8 medical centers. Among the included population, male patients accounted for 87.8%, with 75.6% in advanced stages. Notably, 46.3% of cases involved bone marrow, while 19.5% involved the central nervous system (CNS). The most commonly used chemotherapy regimen was DA-EPOCH ± R, accounting for 53.6% of cases. The overall response rates for patients receiving DA-EPOCH ± R and R-Hyper-CVAD were 59% and 58.2%, respectively. Interestingly, patients receiving regimens containing rituximab had similar complete remission rates (25% vs. 23.5%) and overall survival time (45.69 ± 11.58 vs. 47.79 ± 11.72 months, *P* = 0.907) compared to those without rituximab, but differed in progression rates (33.3% vs. 47.1%). For the entire cohort, the 1-year progression-free survival (PFS) and overall survival (OS) rates were 52% and 67%, respectively. CNS involvement was independent risk factors for survival, with 1-year PFS and OS rates of 0% and 38% for patients with CNS involvement, and PFS and OS rates of 66% and 75% for patients without CNS involvement.

**Conclusions:**

HIV-associated BL patients in China have poor prognosis and show limited response to current treatment regimens. The absence of CNS involvement significantly improves clinical outcomes. The use of rituximab is not significantly associated with improved outcomes but can reduce disease progression.

## Introduction

Burkitt lymphoma (BL) is a highly aggressive B-cell non-Hodgkin lymphoma (NHL) that accounts for 1–2% of NHL cases [1]. Among them, immunodeficiency-related BL, especially HIV-related BL, is one of the most significant types. Generally, HIV-related lymphomas have a higher tumor burden, more extranodal involvement, elevated LDH levels, and a tendency to involve the reproductive system [2, 3]. Compared to other HIV-related lymphomas, such as diffuse large B-cell lymphoma, the incidence of BL in HIV-positive individuals has not decreased after receiving antiretroviral therapy (ART) [4].

Before the era of antiretroviral therapy, many patients died early due to opportunistic infections. However, over the past two decades, there have been significant changes in the treatment strategies for BL patients, such as the use of intensified treatment regimens [5–7] and the addition of rituximab [8]. Patients with a CD4 count above 50 cells per microliter can safely use rituximab [9].

The prognosis of BL has improved, but compared to Western patients, this subset of patients in China may exhibit different clinical behaviors and treatment outcomes. To gain a deeper understanding of HIV-related BL and improve its diagnosis and treatment strategies, The Central and Western China AIDS Lymphoma League emerged. In this study, we retrospectively analyzed the clinical characteristics and prognostic factors of HIV-related BL patients from multiple centers in China.

## Method

### Patients

A retrospective analysis was conducted on 41 HIV-related BL patients who were treated at 8 medical centers affiliated with The Central and Western China AIDS Lymphoma League between April 2012 and April 2021. The diagnosis was based on the hematological and lymphoid tissue tumor World Health Organization (WHO) classification criteria [10], and the diagnosis of all patients was independently reviewed by two expert pathologists. Patients who did not receive lymphoma treatment were excluded from the study. All patients were confirmed to have HIV infection at the time of lymphoma diagnosis. The institutional review boards of each participating center approved the study, which followed the guidelines of the Helsinki Declaration. Written informed consent was obtained from the patients prior to the study.

### Clinical data analysis

The researchers collected comprehensive demographic data (sex, age, household registration) of the patients, CD4 and CD8 cell counts at lymphoma diagnosis, Eastern Cooperative Oncology Group (ECOG) performance status, serum LDH levels, Ann Arbor staging, extranodal involvement, bone marrow involvement, central nervous system (CNS) involvement, presence of bulky tumor (maximum diameter ≥ 7.5 cm), and other viral infections. Serum lactate dehydrogenase (LDH) levels were standardized according to the institutional upper limit of normal (ULN). All patients were assessed using the BL-IPI scoring system and categorized into low, intermediate, and high-risk groups [11].

### Treatment regimens and evaluations

The Hyper-CVAD regimen consists of cyclophosphamide 300 mg/m^2^ every 12 h on days 1–3, dexamethasone 40 mg/d on days 1–4 and 11–14, vincristine 1.4 mg/m^2^ on days 4 and 11, and doxorubicin 50 mg/m^2^ on day 4. The MA regimen includes methotrexate 1 g/m^2^ on day 1 and cytarabine 2 g/m^2^ every 12 h on days 2–3. The DA (dose-adjusted)-EPOCH regimen consists of etoposide 50 mg/m^2^ on days 1–4, doxorubicin 10 mg/m^2^ on days 1–4, vincristine 0.4 mg/m^2^ on days 1–4, oral prednisone 60 mg/m^2^ on days 1–5, and cyclophosphamide 750 mg/m^2^ on day 5. In addition, rituximab is administered at a dose of 375 mg/m^2^.

All patients receive cART treatment, which includes two nucleoside reverse transcriptase inhibitors and one non-nucleoside reverse transcriptase inhibitor. Treatment response is determined by clinical physicians at each institution through PET-CT or whole-body CT evaluations.

### Statistical analysis

Follow-up was conducted through telephone or electronic medical records systems, with a cut-off date of November 2021. Overall survival (OS) was defined as the time from diagnosis to the last follow-up or death from any cause. Progression-free survival (PFS) was defined as the time between diagnosis and disease progression, disease recurrence, or death from any cause. Univariate and multivariate Cox regression analyses were performed to determine potential risk factors for mortality. All statistical analyses were conducted using GraphPad Prism 9. Survival rates were estimated using the Kaplan-Meier curve and compared using the log-rank test. A *p*-value < 0.05 was considered statistically significant.

## Results

### Clinical features

In our study, the median age of patients was 40 years (range: 21 to 72 years). The majority of patients were male (87.8%) and most were in the advanced stage (75.6%). Nearly half of the patients had bone marrow involvement, and 19.5% had central nervous system involvement. 48.8% (20/41) of patients had 3x elevated serum lactate dehydrogenase (LDH) levels. 24.4%of patients had extranodal involvement. Additionally, the median CD4 count was calculated as 245 cells/μl (range: 9 to 729 cells/μl). According to the BL-IPI score, 29.2% of patients in this cohort were classified as high risk. Table 1 provides detailed information on their clinical characteristics.

**Table 1:** Clinical features of patients with HIV-associated Burkitt lymphoma at diagnosis (n = 41)

Features	N (%)
Sex	
Male	36 (87.8)
Female	5 (12.2)
Age	
<40	20 (48.8)
≥40	21 (51.2)
Household registration	
Rural	13 (31.7)
Urban	28 (68.3)
Marital state	
Single	15 (36.6)
Being married	24 (58.5)
Divorced/widowed	2 (4.9)
Primary site	
Nodal	31 (75.6)
Extra-nodal	10 (24.4)
Ann Arbor stage	
I-II	10 (24.4)
III-IV	31 (75.6)
B symptom	
Presence	26 (63.4)
Absence	15 (36.6)
ECOG PS	
0–1	25 (61.0)
≥2	16 (39.0)
BM involvement	
Yes	19 (46.3)
No	22 (53.7)
CNS involvement	
Yes	8 (19.5)
No	33 (80.5)
Bulky disease	
Yes	14 (34.1)
No	27 (65.9)
CD4 count	
≤100cells/μl	5 (12.2)
>100cells/μl	36 (87.8)
LDH level	
≤ 3x ULN	21 (51.2)
>3x ULN	20 (48.8)
Other viral infections	
Epstein-Barr virus	16(39.0)
Hepatitis B	3(7.3)
Hepatitis C	0(0)
BL-IPI staging	
Low	7 (17.1)
Intermediate	22 (53.7)
High	12 (29.2)
First-line treatment	
R-Hyper-CVAD/MA	12 (29.3)
R-DA-EPOCH	12 (29.3)
DA-EPOCH	10 (24.3)
Others	7 (17.1)
Rituximab	
With	24 (58.5)
Without	17 (41.5)

BM, bone marrow; CNS, central nervous system; ECOG PS, Eastern Cooperative Oncology Group performance status

### Treatment and response

58.5% of patients received chemotherapy based on rituximab. The most commonly used chemotherapy regimen was DA-EPOCH ± R (53.6%), which included R-DA-EPOCH (29.3%) and DA-EPOCH (24.3%). Only one patient received the CODOX-M/IVAC treatment regimen. The overall response rate (ORR) to first-line treatment in the entire cohort was 53.7%, with a complete response rate (CR) of 24.3% and a partial response rate (PR) of 29.2%. The CR and PR rates for patients receiving the DA-EPOCH ± R regimen were 36.3% and 22.7% respectively, while the CR and PR rates for patients receiving the R-Hyper-CVAD/MA regimen were 16.6% and 41.6% respectively. The complete response rate for patients receiving rituximab-containing regimens was similar to that of patients not receiving rituximab (25% vs. 23.5%). Only 4 patients (9.7%) underwent hematopoietic stem cell transplantation.

Only 11 patients (11/41, 26.8%) received systemic methotrexate and cytarabine therapy, while 30 patients (30/41, 73.2%) received intrathecal injections as central nervous system prophylaxis. Among the 8 HIV-associated Burkitt lymphoma patients with central nervous system involvement at initial diagnosis, one achieved complete remission after 4 cycles of EPOCH treatment, but later experienced rapid disease progression.

Additionally, 41% of patients experienced disease progression. Patients treated with rituximab had a lower risk of disease progression compared to those not treated with rituximab (33.3% vs. 47.1%). Among patients treated with the DA-EPOCH ± R regimen, 36.4% (8/22) experienced disease progression, while 41.7% (5/12) of patients treated with the R-Hyper-CVAD/MA regimen experienced disease progression.

### Survival and prognostic factors

**Fig. 1:**
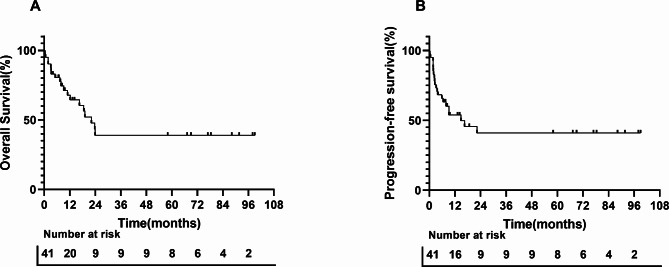
Overall survival (**A**) and progression-free survival (**B**) for patients with HIV-associated Burkitt lymphoma

**Fig. 2:**
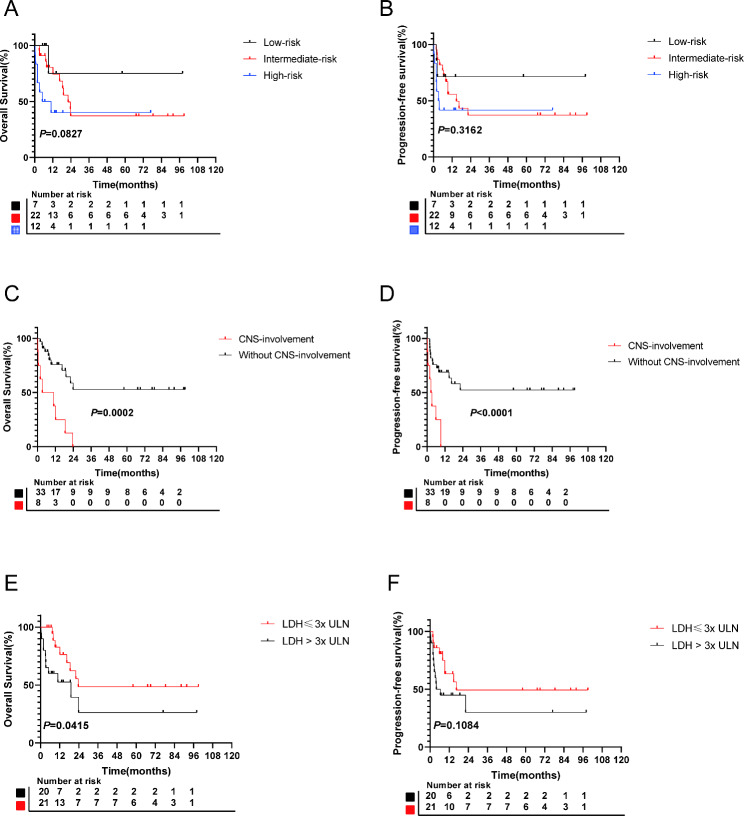
The prognosis of patients with HIV-associated Burkitt lymphoma. The overall survival (**A**) and progression-free survival (**B**) of low-, intermediate- and high-risk subgroups. The overall survival (**C**) and progression-free survival (**D**) of central nervous system involvement. The overall survival (**E**) and progression-free survival (**F**) of patients with LDH > 3x ULN. ULN, upper limit of normal

In this study, 17.1% (7/41) of patients were lost to follow-up. The median follow-up period was 10.9 months (range 0.3–99 months). At the last follow-up, 46.3% (19/41) of HIV-associated Burkitt lymphoma patients had died. Disease progression was the main cause of death (8/19, 42.1%), followed by infection (7/19, 36.8%). The 1-year progression-free survival rate and overall survival rate for the entire cohort were 52% and 67% respectively (Fig. 1). According to the Burkitt lymphoma International Prognostic Index (BL-IPI) score, the 1-year overall survival rates for the low-risk, intermediate-risk, and high-risk groups were 75%, 62%, and 40% respectively (Fig. 2A). Additionally, the 1-year progression-free survival rates for the low-risk, intermediate-risk, and high-risk groups were 67%, 55%, and 39% respectively (Fig. 2B). The choice of first-line treatment (based on the DA-EPOCH regimen or R-Hyper-CVAD/MA regimen) did not significantly affect survival outcomes, whether in terms of overall survival (*P* = 0.821) or progression-free survival (*P* = 0.789).

Table 2 shows the results of the univariate and multivariate analyses in HIV-associated Burkitt lymphoma patients. Both the univariate and multivariate analyses showed a significant association between LDH level, central nervous system (CNS) involvement, and survival. However, in multivariate analysis, only CNS involvement was identified as an independent risk factor for predicting progression-free survival in HIV-associated Burkitt lymphoma patients. The 1-year overall survival rate and progression-free survival rate for patients with CNS involvement were 38% and 0%, respectively, compared to 75% and 66% for those without involvement (Fig. 2C-D). The 1-year overall survival rate for patients with LDH levels exceeding the upper limit of normal (ULN) threefold was 50%, while those without this condition had a rate of 84% (Fig. 2E). The 1-year progression-free survival rate for patients with LDH levels exceeding the ULN threefold was 41%, while those without this condition had a rate of 63% (Fig. 2F). In addition, the analysis of overall survival time with or without rituximab showed no significant difference, with durations of 45.69 ± 11.58 and 47.79 ± 11.72, respectively (*P* = 0.907).

**Table 2:** Risk factors for survival in patients with HIV-associated Burkitt lymphoma

	Overall survival				Progression-free survival			
Variables	Univariate	Multivariate			Univariate	Multivariate		
	*P* value	HR	95%	P* value	*P* value	HR	95%	P* value
Male	0.941				0.826			
Age > 40	0.077				0.277			
Household registration	0.249				0.417			
Primary site	0.668				0.999			
Evaluated LDH	0.889				0.291			
LDH > 3ULN	0.041	2.806	1.050–7.497	0.040	0.108			
ECOG > 1	0.872				0.851			
With B symptom	0.247				0.308			
Ann Arbor stage III-IV	0.835				0.140			
BM involvement	0.397				0.539			
CNS involvement	< 0.001	5.572	2.019–15.375	0.001	< 0.001	5.985	2.165–16.542	0.001
CD4 > 100 cells/μl	0.302				0.522			
HIV plasmaviremia	0.159				0.232			
CD4/CD8 ratio	0.528				0.417			
With bulky disease	0.325				0.136			
Rituximab-containing	0.562				0.392			
Chemotherapy regimens	0.459				0.355			

BM, bone marrow; CNS, central nervous system; ECOG, Eastern Cooperative Oncology Group; * *P* value was calculated by COX regression and *p* < 0.05 regarded significant

### CNS involvement

During the follow-up period, all 8 patients with CNS involvement died. Subsequently, the clinical characteristics of patients with CNS involvement were analyzed. Patients with CNS involvement were more likely to have concurrent B symptoms (100% versus 54.5%, *P* = 0.018) and higher ECOG scores (87.5% versus 27.3%, *P* = 0.003) compared to patients without CNS involvement. The differences in clinical characteristics between the two subgroups are detailed in Table 3.

**Table 3:** Clinical features of patients with HIV-associated Burkitt lymphoma at diagnosis with or without central nervous system involvement

Features	CNS-involvement, N (%)	Without CNS-involvement, N(%)	*P*
Total	8	33	
Sex			
Male	8(100)	28(84.8)	0.563
Age			
<40	6 (75.0)	14(42.4)	0.130
≥40	2 (25.0)	19(57.6)	
Primary site			
Nodal	5 (62.5)	26(78.8)	0.378
Extra-nodal	3 (37.5)	7(21.2)	
Ann Arbor stage			
I-II	0 (0)	10(30.3)	0.165
III-IV	8 (100)	23(69.7)	
B symptom			
Presence	8 (100)	18(54.5)	0.018
Absence	0 (0)	15(45.5)	
ECOG PS			
0–1	1 (12.5)	24(72.7)	0.003
≥2	7 (87.5)	9(27.3)	
BM involvement			
Yes	6 (75.0)	13(39.4)	0.115
No	2 (25.0)	20(60.6)	
Bulky disease			
Yes	2 (25.0)	12(36.4)	0.692
No	6 (75.0)	21(63.6)	
CD4 count			
≤100cells/μl	2 (25.0)	3(9.1)	0.246
>100cells/μl	6 (75.0)	30(90.9)	
LDH level			
≤ 3x ULN	3 (37.5)	18(54.5)	0.454
>3x ULN	5 (62.5)	15(45.5)	
BL-IPI staging			
Low	0(0)	7(21.2)	0.08
Intermediate	4 (50.0)	18(54.5)	
High	4 (50.0)	8(24.2)	
First-line treatment			
R-Hyper-CVAD/MA	3 (37.5)	10(30.3)	0.505
R-DA-EPOCH	3 (37.5)	9(27.3)	
DA-EPOCH	1 (12.5)	9(27.3)	
Others	1 (12.5)	5(15.1)	
Rituximab			
With	5 (62.5)	19(57.6)	0.563
Without	3(37.5)	14(42.4)	

BM, bone marrow; CNS, central nervous system; ECOG PS, Eastern Cooperative Oncology Group performance status

## Discussion

To our knowledge, this is the first multicenter report in China on the clinical characteristics and prognosis of HIV-associated Burkitt lymphoma.

Burkitt lymphoma is a rare form of non-Hodgkin lymphoma with consistently poor clinical outcomes, particularly in HIV-positive patients. In a recent cohort of 246 HIV-positive Burkitt lymphoma patients from the United Kingdom and the United States [12], a 3-year progression-free survival rate and overall survival rate of 61% and 66% were achieved, whereas in our study, the 1-year PFS rate and OS rate were only 52% and 67%. A multicenter trial evaluating the R-DA-EPOCH regimen in 28 untreated HIV-positive Burkitt lymphoma patients showed a 4-year event-free survival rate of 84.9% and overall survival rate of 87% [8]. Thirteen HIV-related Burkitt lymphoma or leukemia patients treated with Hyper CVAD had a 2-year overall survival rate of 48% and a complete response rate of 92% [13]. A study from Japan reported an overall response rate of 78.8% and a 2-year overall survival rate of 72.6% in 23 patients treated with Hyper CVAD [14]. In our study, half of the patients received DA-EPOCH-based chemotherapy, but the overall response rate was only 59%, which is unsatisfactory. Similarly, the overall response rate for patients treated with R-Hyper-CVAD was only 58.2%. A meta-analysis of 646 patients demonstrated that the use of rituximab improved overall survival, although the differences in most clinical trials were not significant [15]. The use of rituximab remains controversial. We also found that the use of rituximab was not significantly associated with improved clinical outcomes in patients, although it may reduce disease progression to some extent. The existing treatment regimens have limited efficacy for patients in China, and further clinical trials are needed to explore more appropriate treatment strategies.

The incidence of CNS involvement in patients in this study was consistent with previous publications [13, 14, 16, 17]. In univariate and multivariate analysis, CNS involvement was identified as an independent risk factor for prognosis in Burkitt lymphoma patients, with all 8 patients with CNS involvement dying within 2 years. Half of the patients received DA-EPOCH-based regimens, followed by R-Hyper-CVAD (37.5%), and one patient received CHOP regimen, but died due to concurrent infection after disease progression. In a multicenter study involving 641 Burkitt lymphoma patients, CNS involvement was found to be associated with worse prognosis regardless of the first-line treatment used, and was not influenced by other factors such as HIV infection [18]. Therefore, finding strategies more suitable for the treatment of CNS involvement is one of the important research goals for the future.

Due to the retrospective nature of this multicenter study, there is a paucity of data on drug dose adjustment and partial HIV treatment, and treatment decisions varied considerably among the different centers. The outcomes observed in our cohort were disheartening, with a majority of patients succumbing to infectious or progressive diseases. To improve clinical outcomes, it will be imperative to focus on preventing concurrent infections, increasing the utilization of rituximab, and developing novel therapies tailored to the Asian population.

## Conclusion

The prognosis for HIV-associated Burkitt lymphoma patients in China is bleak, with current treatment regimens demonstrating low response rates. There is a significant correlation between improved outcomes and LDH ≤ 3ULN levels as well as the absence of central nervous system involvement. Although the administration of rituximab did not show a significant correlation with improved outcomes, it has been found to reduce disease progression rates.

## Data Availability

The authors state that the manuscript contains the data supporting the study’s conclusions.
